# Classical and Non-classical Presentations of Complement Factor I Deficiency: Two Contrasting Cases Diagnosed via Genetic and Genomic Methods

**DOI:** 10.3389/fimmu.2019.01150

**Published:** 2019-06-07

**Authors:** Adrian M. Shields, Alistair T. Pagnamenta, Andrew J. Pollard, OxClinWGS, Jenny C. Taylor, Jenny C. Taylor, Holger Allroggen, Smita Y. Patel

**Affiliations:** ^1^Clinical Immunology Service, Institute of Immunology and Immunotherapy, University of Birmingham, Birmingham, United Kingdom; ^2^Department of Clinical Immunology, John Radcliffe Hospital, Oxford, United Kingdom; ^3^NIHR Oxford Biomedical Research Centre, Wellcome Centre for Human Genetics, University of Oxford, Oxford, United Kingdom; ^4^Oxford Vaccine Group, Department of Paediatrics, University of Oxford, Oxford, United Kingdom; ^5^Department of Neurology, University Hospital Coventry, Coventry, United Kingdom

**Keywords:** complement factor I, primary immunodeficiency, genomic medicine, pneumococcal infection, neuroinflammation, complement deficiency

## Abstract

Deficiency of complement factor I is a rare immunodeficiency that typically presents with increased susceptibility to encapsulated bacterial infections. However, non-infectious presentations including rheumatological, dermatological and neurological disease are increasingly recognized and require a high-index of suspicion to reach a timely diagnosis. Herein, we present two contrasting cases of complement factor I deficiency: one presenting in childhood with invasive pneumococcal disease, diagnosed using conventional immunoassays and genetics and the second presenting in adolescence with recurrent sterile neuroinflammation, diagnosed via a genomic approach. Our report and review of the literature highlight the wide spectrum of clinical presentations associated with CFI deficiency and the power of genomic medicine to inform rare disease diagnoses.

## Introduction

The human complement system is a tightly regulated cascade of soluble and membrane-bound proteins that maintains immune homeostasis by clearing apoptotic cells and immune complexes, and facilitates host defense via the opsonization and direct killing of microbial pathogens. Monogenic deficiencies of complement factors can result in susceptibility to infection by encapsulated bacteria and, in some cases, systemic lupus erythematosus-like disease, glomerulonephropathies and vasculitides.

Complement factor I (CFI) is an 88 kDa glycoprotein that is principally produced by the liver. It circulates in a zymogen-like state prior to activation by furin (1), which cleaves a linker from CFI to produce a heterodimeric protein formed of a 55 kDa heavy chain and 37 kDa light chain joined by a single disulphide bond. CFI is recruited to sites of complement deposition where regulatory proteins act as co-factors for its enzymic activity. When acting with a co-factor protein (complement factor H, CD46, C4 binding protein, or CD35), CFI binds and sequentially cleaves C3b into iC3b, C3c, C3dg, and finally C3d (2). CFI and can similarly cleave C4b. The cleavage products of C3 and C4 lack the capacity to form C3 convertases that further amplify the complement cascade but continue to facilitate the removal of immune complexes. Thus, CFI acts as an essential regulator of classical and alternative complement cascade activation.

The understanding of the spectrum of disease associated with deficiency of complement factor I has broadened considerably in recent years. Herein, we report two contrasting cases of complement factor I deficiency; one presenting with spontaneous pneumococcal peritonitis in childhood and the other presenting with recurrent, sterile neuroinflammation in adolescence.

## Case 1

**Figure 1:**
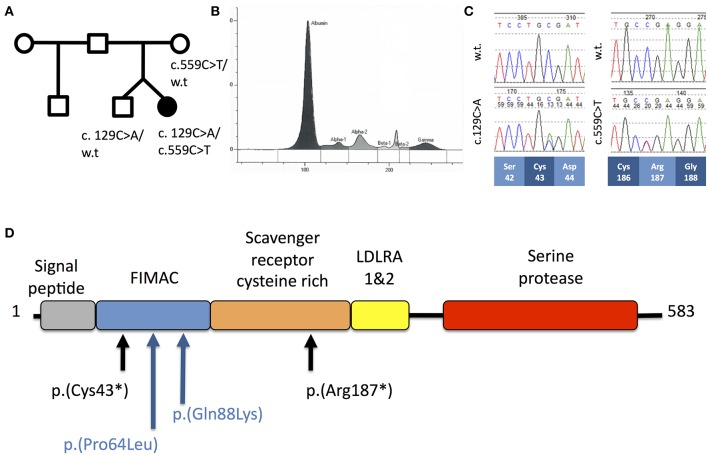
**(A)** Pedigree of Patient A. **(B)** Capillary zone electrophoresis trace from Patient A demonstrating absent beta-2 peak. **(C)** Sequencing chromatograms from Patient A demonstrating c.129C>A and c.559C>T mutations in the heterozygous states. Codon positions and wild-type amino-acids are indicated below. **(D)** Schematic of the domain structure of complement factor I protein demonstrating position of mutations from Patient A (black) and Patient B (blue).

A 2-year-old girl (patient A) presented to the emergency department with a 24-h history of lethargy, fever and abdominal pain. One week previously, she had suffered from a mild gastroentritis-like illness but had recovered fully. She was a dichorionic-diamniotic twin born at 33 weeks, was previously well and fully vaccinated according to the UK schedule, including neonatal BCG. There was no family history of immunodeficiency (Figure 1A).

On admission, she had a distended abdomen, tachycardia, pyrexia (39°C) and raised inflammatory markers; she was admitted and treated as suspected appendicitis. At laparotomy, frank pus was found in the abdomen but the appendix appeared grossly normal. A diagnosis of spontaneous bacterial peritonitis was made. Blood cultures from admission grew serotype 10A *S. pneumoniae*, a strain not contained within the 13-valent pneumococcal conjugate vaccine administered to children in the UK.

Routine investigation of pediatric invasive pneumococcal disease in our center is based on the protocol described by Gashinard et al. (3). The patient's results are summarized in Table 1 and Supplementary Table 1. The beta-2 peak on serum electrophoresis was absent, commensurate with low C3 (Figure 1B). Significantly reduced activity of both the classical and alternative complement pathways was noted and subsequent investigation demonstrated completely absent CFI and reduced levels of complement factors B and H indicative of consumption.

**Table 1:** Summary of clinical and immunological characteristics of published, genetically confirmed cases of CFI deficiency.

**Case**	**Age at first presentation (yr)**	**Family**	**Gender**	**Origin**	**Allele 1**	**Allele 2**	**Protein 1**	**Protein 2**	**Domain 1**	**Domain 2**	**Infections**	**Other manifestations**	**CFI**	**CFH**	**CFB**	**C3**	**AP50**	**CH50**	**References**	**Notes**
1	10	1	F	British	c.191C>T	c.262C>A	p.P64L	p.Q88K	FIMAC	FIMAC		Recurrent haemorrhagic leukoencephalitis	Undetectable	n/a	n/a	0.43 g/l	<50%	876 (1,000–2,000)	This publication	
2	n/a	2	F	Turkish	c.162C>G	Homozygous	p.C54W	Homozygous	FIMAC	FIMAC		Leukocytoclastic vasculitis	<5%	47.00%	<3%	0.29 g/L	0.00%	21.00%	(4)	
3	2	3	M	Australian	c.133-134delAA	Homozygous	p.K45Sfs^*^11	Homozygous	FIMAC	FIMAC	Recurrent infections with *S. pneumoniae* and *S. pyogenes* including septicaemia, joint infections and pneumonia	Juvenile idiopathic arthritis	Undetectable	189 mg/L (345–590)	<38 mg/L (191–382)	0.32 g/L (0.7–2.06)	Undetectable	41.00%	(5)	
4	Childhood	3	M	Australian	c.133-134delAA	Homozygous	p.K45Sfs^*^11	Homozygous	FIMAC	FIMAC	Otitis media		Undetectable	148 mg/L (345–590)	<38 mg/L (191–382)	0.22 g/L (0.7–2.06)	Undetectable	11.00%	(5)	
5	n/a	4	F	Spanish	c.80_81delAT	c.559C>T	p.D27Afs^*^18	p.R187^*^	FIMAC	SRCR	Meningitis, penumonia	Henoch-Schonlein purpura	0	9.4 mg/dl (12–56)	0 mg/dL (7.5–28)	38.4	n/a	n/a	(6)	
6	10	5	F	Filipino	n/a	n/a	p.G71V	p.C196S	FIMAC	SRCR		Diffuse vasogenic cerebral oedema, neutrophilic brain infiltration	4.6 mcg/ml (29–59)	107 mcg/ml (160–412)	43.8 mcg/ml (127.6–278.5)	41	<10%	21 U/ml (30–75 U/ml)	(7)	Asymptomatic younger sister with same compound heterozygous mutations and complement profile
7	2	6	F	Brazilian/Portuguese	c.129C>A	c.559C>T	p.C43^*^	p.R187^*^	FIMAC	SRCR	Spontaneous pneumococcal peritonitis		Undetectable	211.0 mg/L (345–590)	29.7 mg/L (295–400)	21 mg/dL	21.00%	20.00%	This publication	
8	10	7	M	Filipino	n/a	n/a	p.G71V	Heterozygote	FIMAC			Vasogenic cerebral oedema, neutrophilic cerebral inflammation	6 mcg/ml (29–59)	136 mcg/ml (160–412)	n/a	<40	29.00%	40 U/ml (30–75 U/ml)	(7)	
9	n/a	8	F	Spanish	c.559C>T	c1610_1611insAT	p.R187^*^	p.V537Vfs^*^2	SRCR	SP	Pneumonia, facial cellulitis	Hypocomplementemic vasculitis	4.00%	4.8 mg/dl (12–56)	0 mg/dL (7.5–28)	31.5	n/a	n/a	(6)	Asymptomatic younger sister with same compound heterozygous mutations and complement profile
10	1 m	9	F	Denmark	c.563G>T	c.1253A>T	p.G188V	p.H418L	SRCR	SP	Recurrent bacterial upper respiratory tract infections, septicaemia, erysipelas		Undetectable	64% (69–154)	<2.5% (59–154)	48.00%	Reduced	Reduced	(8)	
11	Childhood	10	M	Spanish	c.485G>A	Homozygous	p.G162D	Homozygous	SRCR	SRCR	Streptococcus bovis endocarditis, pneumonias, meningitis, sepsis		Undetectable	26.3 mg/dl (12–56)	4.5 mg/dL (20–40)	29.6	0.00%	<12.1 U/ml	(9)	
12	n/a	11	M	Spanish	c. 772 G>A	c. 772 G>A	p.D220-K257del	p.D220-K257del	LDRA1	LDRA1	Pneumonia, meningococcal septicaemia, oral thrush, balanitis		0.00%	8.40 mg/dl (12–56)	0 mg/dL (7.5–28)	22.6	n/a	n/a	(6)	
13	16	12	F	Spanish	c.739T>G	Homozygous	p.C247G	Homozygous	LDRA1	LDRA1	Recurrent meningitis coinciding with menstruation		n/a	52 mcg/ml (200–600)	6.7 mg/dL (17–60)	24 mg/dl	n/a	<50 U/ml	(10, 11)	
14	18	12	F	Spanish	c.739T>G	Homozygous	p.C247G	Homozygous	LDRA1	LDRA1	Meningitis, recurrent tonsillitis		3.00%	65 mcg/ml (200–600)	7.3 mg/dl (17–60)	22 mg/dl	n/a	<50 U/ml	(10, 11)	
15	2	13	F	Spanish	c.772G>A	Homozygous	p.D220-K257del	Homozygous	LDRA1	LDRA1	Meningococcal meningitis, pneumococcal meningitis	Hyperpigmented skin lesions	n/a	100 mcg/ml (200–600)	<12 md/dL (17–60)	16.5 mg/dl	n/a	140 U/ml (200–400 U/ml)	(10)	
16	31	13	M	Spanish	c.772G>A	Homozygous	p.D220-K257del	Homozygous	LDRA1	LDRA1	Lymphoid meningitis	Hyperpigmented skin lesions	n/a	80 mcg/ml (200–600)	n/a	24.7 mg/dl	n/a	136 U/ml (200–400 U/ml)	(10)	
17	9	13	M	Spanish	c.772G>A	Homozygous	p.D220-K257del	Homozygous	LDRA1	LDRA1	Otitis, septic arthritis	Hyperpigmented skin lesions	n/a	60 mcg/ml (200–600)	<12 md/dL (17–60)	17.3 mg/dl	n/a	142 U/ml (200–400 U/ml)	(10)	
18	4	14	F	Turkish	c.764G>A	Homozygous	p.C255Y	Homozygous	LDRA1	LDRA1	Recurrent upper and lower respiratory tract infections, meningitis	Recurrent vasculitic eruptions, immune complex glomerulonephritis, microscopic haematuria	Undetectable	48% (69–154)	<12% (59–154)	0.48 g/L (0.77–1.38)	Undetectable	Normal range	(8, 12)	2 female siblings share genotype—disease manifestations not reported; 1 female sibling died of sepsis at 18 m but DNA was not available
19	n/a (diagnosed at 23)	15	F	Swedish	c.748C>A	c.803C>T	p.Q250K	p.S268K	LDRA1	LDRA2		Systemic lupus erythematosus	2.00%	85% (69–154)	44% (59–154)	63.00%	Reduced	Normal range	(8)	
20	10	16	F	Croatia	c.772G>A	c.1100T>G	p.D220-K257del	p.I357M	LDRA1	SP	Pneumonia, recurrent upper respiratory tract infections		Undetectable	81% (69–154)	13% (59–154)	73.00%	Normal	Reduced	(8)	
21	n/a (diagnosed at 18)	17	F	Turkish	c.866A>T	Homozygous	p.D289V	Homozygous	LDRA2	LDRA2	Recurrent upper and lower respiratory tract infections	Recurrent vasculitic eruptions and arthralgias	Undetectable	65% (69–154)	~10% (59–154)	0.47 g/L (0.7–2.06)	Undetectable	13.00%	(5)	
22	5	18	M	Spanish	c.1420 C>T	5.6 kB gene deletion	p.R474^*^	-	SP	-	Meningitis with meningococcal septicaemia, otitis		0.00%	19.5 mg/dl (12–56)	0 mg/dL (7.5–28)	33.4	n/a	n/a	(6)	Asymptomatic younger brother with same compound heterozygous mutations and complement profile
23	4 m	19	F	UK	c.1253A>T	c.772G>A	p.H418L	p.D220-K257del	SP	LDRA1	Pneumococcal meningitis, recurrent meningococcal meningitis, otitis media		Undetectable	n/a	10.00%	30.00%	Undetectable	14 U/ml (28–45 U/ml)	(13, 14)	
24	2	20	F	Pakistani	c.1139A > G	Homozygous	p.H380R	Homozygous	SP	SP	Otitis media, lower respiratory tract infection	Cutaneous vasculitis, arthralgia	36% (19 mg/L)	219 mg/L (36%)	n/a	22	0.00%	5.00%	(15)	Asymptomatic older brother with same homozygous mutations and complement profile
25	16	21	F	Belgian	c. 1019 T>C	c. 1571 A>C	p.I340T	p.D524V	SP	SP		Aseptic meningoencephalitis, leukocutaneous vasculitis	44 mg/L (25–44)	460 mg/L (360–680)	1 mg/dL (8–21)	57	0.00%	97.00%	(16)	
26	4	22	F	Pakistani	c.1139A>G	Homozygous	p.H380R	Homozygous	SP	SP	Otitis media	Recurrent abdominal pain	2.5 mg/dL	35.5 mg/dl (12–56)	1.2 mg/dL (20–40)	35.2	0.00%	<12.1 U/ml	(9)	Asymptomatic younger brother with same homozygous mutation, absent factor I but normal C3
27	18 m	23	M	Scottish	c.1253A>T	Homozygous	p.H418L	Homozygous	SP	SP	Staphylococcus epidermidis septic arthritis, meningococcal meningitis, recurrent sinusitis, facial cellulitis		Undetectable	46.00%	Undetectable	28.00%	Undetectable	Undetectable	(13, 17)	Asymptomatic older sister with same homozygous mutation
28	Childhood	24	M	Spanish	c.1450_1454delCTTCA	Homozygous	p.L484Vfs^*^3	Homozygous	SP	SP	Otitis media, pharyngitis, invasive meningococcal infection, infected sacral cyst		Undetectable	19.14 mg/dL (12–56)	0.77 mg/dL (20.5–40)	19.3 mg/dl	Undetectable	2 UI/ml (34–71 UI/ml)	(18)	
29	15 m	25	F	Brazilian	c.1176insAT	Homozygous	p.W393Yfs^*^5	Homozygous	SP	SP	Post-operative infection, bacterial meningitis, otitis, pneumonia	Henoch-Schonlein purpura and subsequent systemic lupus erythematosus: diffuse proliferative membranous glomerulonephritis, psychosis, seizures, stroke, photosensitive malar rash	Undetectable	93 (454 ± 124 mcg/ml)	Undetectable	127 ug/ml (1,300–1,500)	Undetectable	Undetectable	(19, 20)	
30	3	25	F	Brazilian	c.1176insAT	Homozygous	p.W393Yfs^*^5	Homozygous	SP	SP	Adenoid hyperplasia, gastrointestinal infection progressing to fatal severe bilateral pneumonia		Undetectable	105 (454 ± 124 mcg/ml)	Undetectable	259 ug/ml (1,300–1,500)	Undetectable	Undetectable	(19, 20)	
31	3 m	26	F	Argentinian	c.1006C>T	Homozygous	p.Q354^*^	Homozygous	SP	SP	Otitis media, recurrent pneumonia	Recurrent vasculitis	Undetectable	100% (69–154)	<12% (59–154)	39.00%	Reduced	Reduced	(8)	
32	4	27	F	Spanish	c.1176_1177dupAT	c.485G>A	p.W393Yfs^*^5	p.G162D	SP	SRCR	Otitis, sinusitis, bronchitis, meningococcal septicaemia	Arthritis	2.00%	8.23 mg/dl (12–56)	0 mg/dL (7.5–28)	22.8	n/a	n/a	(6)	

*FIMAC, factor I membrane attack complex; SRCR, scavenger receptor cysteine rich; SP, serine protease; LRDA, low density lipoprotein receptor class A domain*.

The clinical diagnosis was confirmed by Sanger sequencing of *CFI* (NM_000204.4) in the proband, which revealed compound heterozygous variants (c.129C>A; p.Cys43^*^ and c.559C>T; p.Arg187^*^, Figure 1C) predicting protein truncation within the factor I membrane attack complex (FIMAC) domain and scavenger receptor cysteine rich domain, respectively (Figure 1D). The p.Cys43^*^ variant has not previously been reported, however the p.Arg187^*^ variant has been identified in two individuals with complete CFI deficiency, on each occasion in trans with a frameshifting allele (Table 1). The p.Arg187^*^ variant has an allele frequency of 0.00001415 with no homozygote identified in gnomAD.

The patient remains well at 5 years and 4 months of age, and has had no further invasive bacterial infections following initiation of prophylactic antibiotics. Vaccination against encapsulated bacteria including *H. influenzae* type b, pneumococcus and meningococcus were optimized with good responses (Supplementary Table 1). Complement levels and function in the proband's twin were normal, excluding complete CFI deficiency, however he was found to be heterozygous for the 129C>A variant.

## Case 2

**Figure 2:**
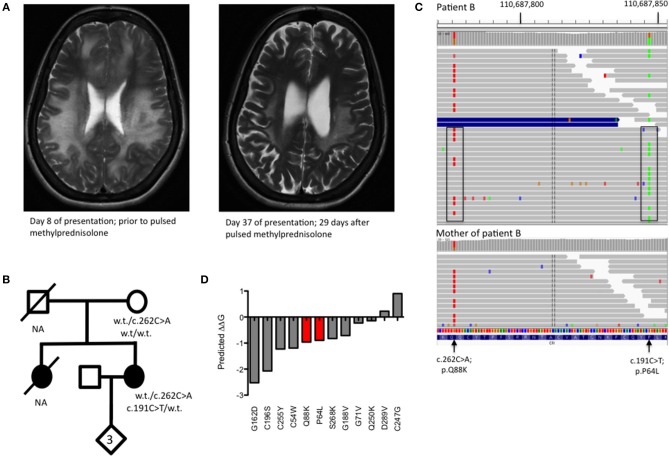
**(A)** T2 weighted MRI imaging from Patient B during acute encephalomyelitis and following treatment with pulsed methylprednisolone. **(B)** Pedigree of Patient B showing genotypes of rare *CFI* variants. NA, DNA not available for deceased individuals. **(C)** Visualization of read-level information shows that the two CFI variants, lying 71 bp apart, are easily phased by the 150 bp reads and are *in trans* (boxed). The absence of the c.191C>T variant in the mother (lower panel) is insufficient to confirm an *in trans* orientation as the variant may have arisen *de novo*. **(D)** Free energy change calculations for known missense, disease-causing point mutations in complement factor I with mutations from Patient B shown in red.

A 32 year old lady (patient B) presented to the emergency department with a 3 day history of gradual onset frontal headache, blurred vision and slurred speech, followed by several tonic-clonic seizures in short succession, deteriorating into coma. Her family reported preceding upper respiratory tract infection symptoms. She was admitted and treated as presumed meningoencephalitis. MRI neuroimaging showed diffuse, confluent cerebral and cerebellar white matter high signal changes, oedema, and mass effect without DWI change (Figure 2A). She had suffered three similar presentations in the past; a severe episode aged 10 and two milder episodes at the ages of 12 and 18. Her sister had died of fulminant haemorrhagic leukencephalopathy at the age of 16 (Figure 2B). The family had not been investigated further.

CSF sampling showed an inflammatory picture (WCC 322, 55% polymorphs), but no bacterial or viral pathogens were detected by routine culture or PCR. C3 was borderline low and acute phase proteins remained normal during her illness. There was no improvement following treatment with empirical antibiotics and antivirals but a slow recovery ensued following pulsed methylprednisolone, with no residual neurological though very mild cognitive deficit. Whole genome sequencing (WGS) was undertaken in the proband and the proband's unaffected mother to achieve a unifying diagnosis. Filtering of all the variants identified by WGS based on quality metrics, deleteriousness, inheritance pattern and biological function led to a short list of 5 genes that were investigated further (Supplementary Figures 1, 2). Of these, the *CFI* gene was the only gene to show the expected compound heterozygosity.

WGS revealed two heterozygous variants in *CFI* (c.191C>T; p.P64L and c.262C>A; p.Q88K) lying within the CFI FIMAC domain (Figure 1D). Although paternal DNA was not available, Illumina read-level information was used to confirm the variants lay *in trans* (Figure 2C). The CADD score for the p.P64L variant is 33.00, SIFT predicted the variant to be damaging and PolyPhen2 predicted the variant to be probably damaging with an allele frequency of 0.0002335; no homozygotes were identified in gnomAD. P64 is highly conserved across taxa (Supplementary Figure 3). Previous reports have associated the p.P64L variant with atypical haemolytic uraemic syndrome (21) and age related macular degeneration (22). The p.Q88K variant has not been reported previously. Although the CADD score for p.Q88K was only 7.34 and SIFT predicted the variant to be tolerated, Polyphen2 predicted the variant to be possibly damaging. Q88 is also highly conserved across taxa (Supplementary Figure 3).

**Figure 3:**
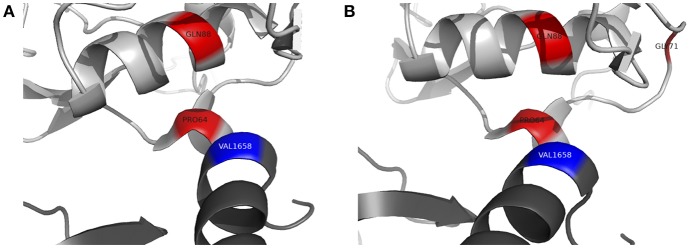
Three-dimensional topology of selected missense mutations in complement factor I (light gray) and their relationship with C3b (dark gray). **(A)** P64 residue of complement factor I forms a contact point with V1658 of C3b (blue). Q88 lies in close apposition to this contact site. **(B)** G71 (*previously reported by Broderick et al and associated with a similar clinical phenotype*) lies on a side chain between P64 and Q88. Figures produced using PyMOL v2.2 using a crystal structure of CFI and C3b solved to a resolution of 4.2 Å (Protein Data Bank Reference – 5O32).

To further assess the pathogenicity of these variants, mutation Cutoff Scanning Matrix (mCSM) analysis was performed (Figure 2D), an approach that predicts the effects of amino acid variation on protein stability by estimating free energy changes (23). Using the CFI crystal structure solved to 2.7 Å (24), mCSM analysis predicted a destabilizing effect of both the p.P64L and p.Q88K variants with ΔΔG of −0.715 and −0.844 kcal/mol, respectively. Three-dimensional modeling of the protein structure of CFI in complex with C3b shows the close topological relationship between the P64 and Q88 residues of CFI and the V1658 residue of C3b (Figure 3A). Furthermore, the G71 residue, mutations of which have also been associated with neurological presentations of CFI deficiency (7), lies on a side chain between these two mutations Figure 3B.

Consistent with these *in silico* prediction, factor I levels were measured in the proband and found to be undetectable. Commensurate reductions in functional activity of the classical and alternative pathway were also identified (Supplementary Table 1) confirming the genomic diagnosis. DNA from the deceased sibling was not available for testing. Heterozygous variants in three other immunologically relevant genes were identified by WGS in Patient B: C6, PTPRC and CD74 (Supplementary Figure 2). The heterozygous variant in C6 illustrates the challenges of interpreting variants based exclusively on bioinformatic predictive scores. Although the CADD score for this variant is 15.1, serum concentrations of the terminal complement complex are elevated in patient B, the assembly of which could not occur without functioning C6. Furthermore, deficiencies in the terminal complement cascade are associated with meningococcal infections, which were not a feature in the clinical presentation. The heterozygous variant identified in PTPRC is unlikely to be clinically relevant given PTPRC variants are associated with severe combined immunodeficiency and the variant was also identified in the healthy mother. The heterozygous variant in CD74 has CADD score of 34; CD74 encodes the class II invariant chain that facilitates peptide loading within the endoplasmic reticulum. Immunodeficiency associated with CD74 variants have not been described.

## Discussion

Complete CFI deficiency is a rare immunodeficiency with 32 genetically characterized cases arising in 27 separate kindreds in the literature (Table 1). By far the most common presentation of complete CFI deficiency is susceptibility to invasive bacterial infections (*S. pneumoniae, N. meningitidis, H. influenzae*) affecting 81.25% of individuals. The remainder present with a range of rheumatological, neurological and dermatological manifestations. Within the genetically confirmed cases, there is a slight female preponderance (2.2:1) and the deficiency typically presents in childhood, although significant diagnostic delay, as seen in Patient B, can occur.

No clear relationship currently exists between mutations in *CFI* and the resulting clinical phenotype of complete CFI deficiency. Factor I activity is dependent on its heavy chain which contains complement regulatory elements, its light chain which contains the serine protease domain, hydrophobic interactions between the heavy and light chain and the presence of a co-factor (e.g., CD35, CD46, or complement factor H) (2, 25). Different co-factors facilitate different patterns of C3b cleavage by CFI (2). The clinical phenotype driven by *CFI* variants will, therefore, depend on how the structural relationships between CFI domains and co-factors are disrupted, whether there is preferential gene expression from different *CFI* alleles, environmental exposure to pathogens and the individual's underlying genetic architecture that may confer susceptibility to infectious or non-infectious manifestations of disease. This is illustrated by the observation that within some of the kindreds described in Table 1, there are siblings that share a *CFI* genotype with an affected proband but remain asymptomatic.

Patient A, who presented with invasive pneumococcal disease in childhood, had biallelic mutations predicted to truncate CFI within the FIMAC and SRCR domains. Neither allele produces a protein with any enzymic function, reflected by the patient's low C3, AP50, and CH50. Susceptibility to encapsulated bacterial infection arises due to a secondary deficiency in C3 and inefficient bacterial opsonization.

The management of infectious complications of complete CFI deficiency is well-established and relies on the optimization of vaccination against encapsulated bacteria and appropriate use of prophylactic antibiotics. As CFI synthesis occurs in the liver, bone marrow transplantation is not a variable strategy to correct CFI deficiency. There is no empirical evidence demonstrating the effectiveness of antibiotic prophylaxis and vaccination in CFI deficiency, however, reports suggest infection frequency reduces with age with some individuals eventually becoming asymptomatic (8, 26). Primary and secondary deficiencies of C3 have been associated with reductions in circulating unswitched and switched memory B cells, without obvious impairment of functional antibody responses (26); close monitoring of functional antibody responses to ensure preservation of immunity against encapsulated organisms is strongly recommended in these patients.

Recurrent sterile neuroinflammation without a significant burden of infection, as seen in patient B, is an unusual presentation of CFI deficiency. The variants identified in patient B are predicted to affect the interface between CFI and C3b. The P64 residue of CFI directly contacts C3b V1658 and forms part of a larger hydrophobic patch on CFI involving residues I55, V60, L63, P64, Y65, F82, P83, and L91 that interacts with the hydrophobic residues V1657, V1658, F1659, and the methylene groups K1570 and K1576 of C3b (2). P64L is predicted to cause steric hindrance at the contact site with C3b. Q88 is a buried residue that forms a hydrogen bond with the carbonyl backbone of T98; the Q88K substitution is predicted to abrogate this interaction however, the precise structural consequences of this substitution are not clear. mCSM analysis predicted a destabilizing effect and three-dimensional modeling suggests Q88 has a close topological relationship with P64. Although CFI was undetectable in patient B's serum, C3 and CH50 were not reduced to the same degree as patient A suggesting that these variants may leave CFI with some residual function.

Four other variants in *CFI* have been associated with severe neuroinflammatory presentations of CFI deficiency. G71V has been identified in the heterozygous state in a patient with acute haemorrhagic leukencephalitis suggesting it may act in a dominant manner (7). G71 lies on a side chain between residues that form the hydrophobic contact site for C3b on CFI. G71V has also been found in the compound heterozygous state with another variant, C196S, hypothesized to disrupt a disulphide bond in the SRCR domain of CFI. The final two variants associated with neurological presentations of CFI deficiency are I340T and D524V (16). These variants lie within the serine protease domain but do not directly affect the CFI catalytic triad of H362, D411 and S507. I340T and D524V *in trans* produce dysfunctional CFI proteins that lacked activity to efficiently regulate alternative complement cascade activation, but did not significantly impact the classical cascade (16).

Together, these cases suggest that mutations that completely abrogate the activity of CFI may lead to presentations dominated by childhood infections but if residual activity is retained, more unusual presentations may arise. Functional assays of CFI activity are challenging given that CFI interacts with multiple cofactors, but may be helpful in resolving the consequences of novel variants identified by genomic technologies (27).

Optimum strategies for the management of neurological presentations of CFI deficiency remain uncertain. Broderick et al described two cases of acute haemorrhagic leukoencephalitis (AHLE) secondary to complete CFI deficiency: a 10 year old female initially responded to high-dose corticosteroids and intravenous immunoglobulin (IVIG) but this was ineffective during a relapse of the condition aged 17. Anakinra significantly improved neurological parameters and radiological appearances on that occasion and was subsequently tapered. The same physicians successfully used a combination of high-dose corticosteroids, IVIG and anakinra to treat a 10 year old with ALHE; weaning of anakinra was not possible due disease relapses (7). Haerynck et al. described the case of a 16 year old girl with recurrent aseptic meningoencephalitis treated with pulsed corticosteroids, initially with good response but with disease relapses failing to respond to plasmapheresis and requiring further treatment with cyclophosphamide and mycophenolate mofetil (16). Direct replacement of CFI using fresh frozen plasma (28) or purified CFI (29, 30) have been attempted historically with transient correction of *in vitro* assays of complement activity. However, long-term clinical outcomes have not been reported and FFP infusions have been associated with anaphylactic reactions (31).

In conclusion, we report two cases of CFI deficiency with quite different presentations and routes taken to diagnosis. Common to both cases was the observation of low C3 and normal C4, indicative of dysregulated complement consumption. Cognizance of this unusual profile of complement tests and the range of presentations of complete CFI deficiency may facilitate timely diagnosis of this rare immunodeficiency.

## Data Availability

All datasets analyzed for this study are included in the manuscript and/or the Supplementary Files.

## Ethics Statement

Full informed consent for publication was obtained from affected individuals or parents prior to the publication of this report. Patient A was enrolled in the study The genetic and functional characterization of patients with Primary Immune Deficiencies, Infections and Inflammatory conditions (South Hampshire Research Ethics Committee, 12/SC/0044) and patient B was enrolled in the study Molecular Genetic and Analysis and Clinical Studies of Individuals and Families at Risk of Genetic Disease approved by the West Midlands Research Ethics Committee, reference 13/WM/0466.

## Author Contributions

AS, AJP, HA, and SP provided clinical care for the individuals described in this manuscript. The Oxford Clinical Whole Genome Sequencing (OxClinWGS) consortium performed the whole genome sequencing. A full list of consortium members is available in the supplementary data. AS, ATP, JT, and SP analyzed the data and wrote the manuscript. All authors provided critical appraisal of the manuscript.

### Conflict of Interest Statement

The authors declare that the research was conducted in the absence of any commercial or financial relationships that could be construed as a potential conflict of interest.

## Abbreviations

AHLE: acute haemorrhagic leukencephalitis
CADD: combined annotation dependent depletion
CFI: complement factor I
CSF: cerebral spinal fluid
DWI: diffusion weighted imaging
gnoMAD: genome aggregation database
FIMAC: factor I membrane attack complex
FFP: fresh frozen plasma
IVIG: intravenous immunoglobulin
mCSM: mutation cutoff scanning matrix
MRI: magnetic resonance imaging
PCR: polymerase chain reaction
PTPRC: protein tyrosine phosphatase receptor C
SIFT: sorting intolerant from tolerant
SRCR, scavenger receptor: cysteine rich
WCC: white cell count
WGS: whole genome sequencing.
