# Supplementary material for: Classical and Non-classical Presentations of Complement Factor I Deficiency: Two Contrasting Cases Diagnosed via Genetic and Genomic Methods
Source: Front Immunol. 2019 Jun 7;10:1150. doi: 10.3389/fimmu.2019.01150 (PMC6568211; doi:10.3389/fimmu.2019.01150)
Supplement: Supplementary file 1 [file Data_Sheet_1.pdf]

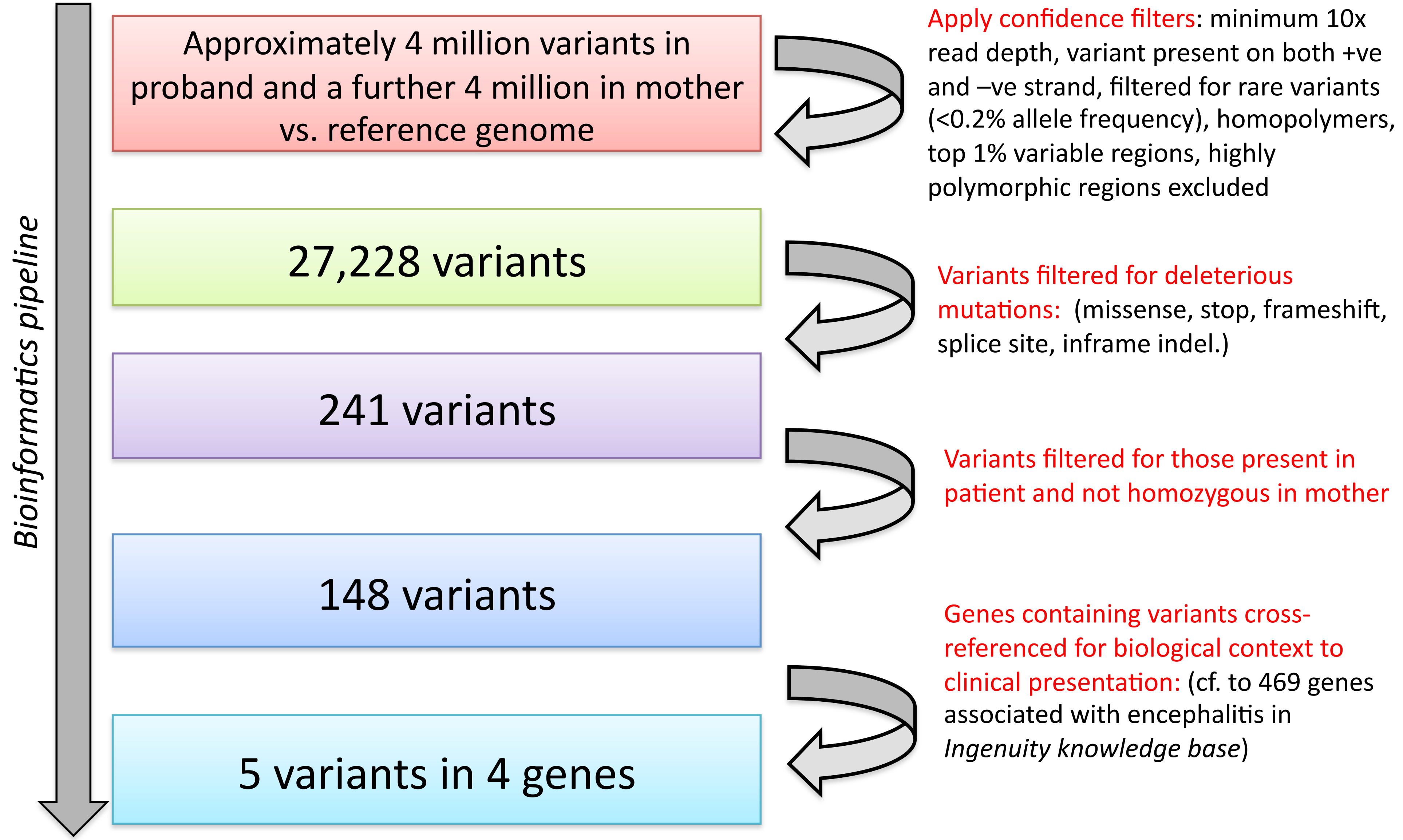

Supplementary Figure 1: Bioinformatics pipeline identifying clinically relevant variants following whole genome sequencing: Illumina 150bp paired-end reads were mapped to hs37d5 using Stampy (V1.0.23) and duplicate reads were marked with Picard (V1.111). Variants were called across both samples simultaneously with Platypus (V0.8.1) using default settings, except for minFlank=0-. Filtering was performed using Ingenuity VA (see www.qiagenbioinformatics.com/products/ingenuity-variant-analysis)

Chr	Position	Ref	Alt	Gene Region	Gene	Comp het	Transcript ID	Transcript Variant	Protein Variant	Patient B - Genotype	Patient B mother - Genotype	SIFT Function Prediction	PolyPhen-2 Function Prediction	CADD Score
**4**	**110,687,776**	**G**	**T**	**Exonic**	***CFI; AC004067.6***	***0***	**NM_001318057.1; ENST00000394634.6; NM_001331035.1; NM_000204.4; ENST00000394635.7; ENST00000645635.1; ENST00000510800.1; ENST00000618244.4; ENST00000512148.5**	**c.262C>A**	**p.Q88K**	**Het**	**Het**	**Tolerated**	**Possibly Damaging**	**< 10**
**4**	**110,687,847**	**G**	**A**	**Exonic**	***CFI; AC004067.6***	***1***	**NM_001318057.1; ENST00000394634.6; NM_001331035.1; NM_000204.4; ENST00000394635.7; ENST00000645635.1; ENST00000510800.1; ENST00000618244.4; ENST00000512148.5**	**c.191C>T**	**p.P64L**	**Het**		**Damaging**	**Probably Damaging**	**33**
5	41,149,482	T	G	Exonic	*C6*	*0*	NM_001115131.2; ENST00000263413.7; NM_000065.3; ENST00000337836.9	c.2484A>C	p.Q828H	Het	Het	Tolerated	Benign	15.08
5	149,782,705	G	A	Exonic; ncRNA; Intronic	*CD74*	*0*	ENST00000009530.11; NR_157074.1; NM_001025159.2; NM_004355.3; ENST00000353334.10; ENST00000377795.7; ENST00000524315.5; NM_001364083.1; NM_001025158.2; NM_001364084.1	c.563-517C>T; c. 538-517C>T; c. 626-517C>T; n. 619C>T; c. 442-517C>T; c. 796C>T; c. 442-1138C>T	p.R266C	Het		Damaging	Probably Damaging	35
1	198,685,960	A	G	Exonic	*PTPRC*	*0*	ENST00000348564.11; NM_080921.3; NM_002838.4; ENST00000442510.7	c.958A>G; c. 1441A>G	p.K320E; p.K481E	Het	Het	Tolerated	Benign	< 10

Supplementary Figure 2: Remaining variants following cross referencing to genes associated with encephalitis in Ingenuity Knowledge Base: Variants in complement factor I are highlighted in red

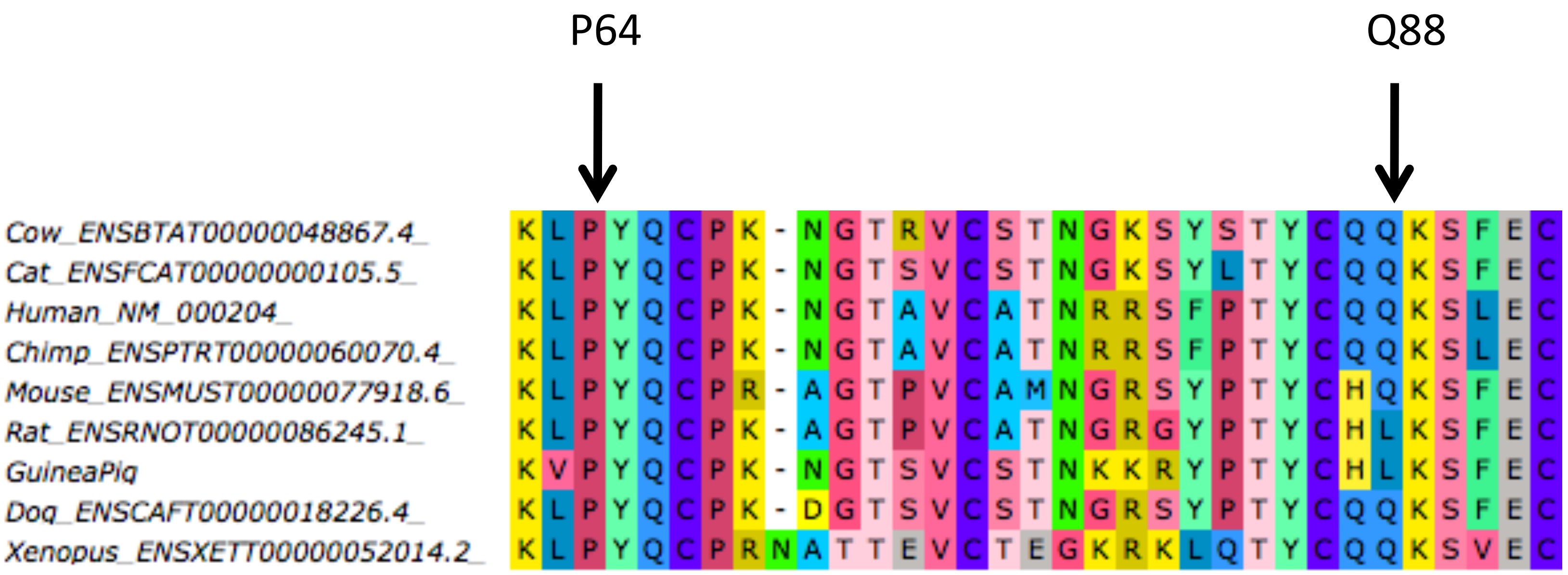

Supplementary Figure 3: Protein sequence alignment of CFI across taxa: The P64 residue and Q88 residue, variants of which were identified in Patient B are highlighted and are highly conserved across taxa.

Supplementary Information: OxClinWGS HICF2 sequencing & analysis consortium

NIHR Oxford Biomedical Research Centre, Wellcome Centre for Human Genetics, University of Oxford, Oxford OX3 7BN, UK

Jenny C. Taylor
Samantha J. L. Knight
Alistair T. Pagnamenta
Niko Popitsch*
Carme Camps
Melissa M. Pentony
Erika M. Kvikstad
Lukas Lange
Mona Hashim
Steve Harris
Mark Tilley
Dimitris Vavoulis
Pamela Kaisaki
Vassilis Ragoussis
Matteo Ferla

*Now at The Children's Cancer Research Institute (CCRI), 1090 Vienna, Austria

Oxford Molecular Diagnostics Centre, Department of Oncology, University of Oxford, Oxford OX3 9DU, UK

Anna H. Schuh
Helene Dreau
Kate E. Ridout
Pauline Robbe
Pavlos Antoniou
Outi Salminen
Maite Cabes
Laura Lopez
Angela Hamblin

Oxford NHS Regional Molecular Genetics Laboratory, Oxford University Hospitals NHS Trust, Oxford OX3 7LE, UK

John Taylor
Conrad Smith

Supplementary Table 1: Results of immunological investigations in patient A and B

	**Patient A**	**Patient B**
C3 (mg/dl)	21.0 (65-190)	43.0 (75-175)
C4 (mg/dl)	26.9 (14-40)	16.0 (14-54)
CH50	27% normal	876 (1000-2000)
AP50	20% normal	Failed to reach 50% of normal control
Terminal complement complex (ng/ml)	2340 (0-80)	331 (0-80)
C5 (mg/L)	46.5 (90-172)	N.D.
C6 (mg/L)	81.6 (45-96)	N.D.
C7 (mg/L)	56.1 (55-85)	N.D.
C8 (mg/L)	103 (40-80)	N.D.
C9 (mg/L)	246 (50-250)	N.D.
Factor H (mg/L)	211 (345-590)	N.D.
Factor I (mg/L)	<2.4 (38-58)	<5.6 (38-58)
Factor B (mg/L)	29.7 (295-400)	N.D.
Serotype specific anti pneumococcal antibodies (protective titer defined as > 0.35mcg/ml)	4/12 at baseline<br>9/12 post Prevenar booster	N.D.
Haemophilus influenza B antibodies	Protective post Menitorix booster	N.D.
Tetanus antibodies	Protective	N.D.
Serum electrophoresis	Absent β2 peak	Normal

N.D. – not determined.
Normal ranges are provided in parenthesis
